# STRIP2, a member of the striatin‐interacting phosphatase and kinase complex, is implicated in lung adenocarcinoma cell growth and migration

**DOI:** 10.1002/2211-5463.12785

**Published:** 2020-02-12

**Authors:** Li‐Min Qiu, Yun‐Hao Sun, Ting‐Ting Chen, Jin‐Jin Chen, Hai‐Tao Ma

**Affiliations:** ^1^ Department of Thoracic Surgery The First Affiliated Hospital of Soochow University Suzhou China; ^2^ Department of Thoracic Surgery Yancheng City No. 1 People's Hospital Yancheng City China; ^3^ Department of Emergency Yancheng City No. 1 People's Hospital Yancheng City China; ^4^ Department of Oncology Yancheng City No. 1 People's Hospital Yancheng City China

**Keywords:** epithelial–mesenchymal transition, invasion, lung adenocarcinoma, migration, proliferation, striatin‐interacting protein 2

## Abstract

Lung adenocarcinoma (LUAD) accounts for ~40% of lung cancer cases, and the 5‐year relative survival rate is no more than 1%. Dysregulation of components of striatin‐interacting phosphatase and kinase (STRIPAK) complexes is associated with various diseases, including cancer. Striatin‐interacting protein 2 (STRIP2), also called Fam40b, has been reported to regulate tumor cell growth and migration. Here, we investigated the role of STRIP2 in LUAD growth, migration and the underlying mechanisms. Analysis of data from The Cancer Genome Atlas database revealed that STRIP2 is highly expressed and predicted poor outcomes in patients with LUAD. Moreover, quantitative RT‐PCR (qRT‐PCR) analysis revealed that the mRNA expression of STRIP2 is greater in all tested LUAD cells than in a normal lung cell line. To investigate the function of STRIP2, we overexpressed STRIP2 in SPC‐A1 cells and depleted STRIP2 in Calu‐3 cells. Cell proliferation was evaluated by Cell Counting Kit‐8 and colony‐forming assays, and Transwell assay was employed to test cell invasion and migration. Our results indicate that STRIP2 depletion suppressed cell proliferation, invasion and migration in Calu‐3 cells, and overexpression of STRIP2 had the opposite effects in SPC‐A1 cells. Moreover, we discovered that STRIP2 depletion reduced the protein levels of p‐Akt and phosphorylated‐mammalian target of rapamycin (p‐mTOR) in Calu‐3 cells, whereas STRIP2 overexpression increased levels of these proteins in SPC‐A1 cells. Furthermore, we found that silencing of STRIP2 clearly enhanced protein levels of E‐cadherin and reduced levels of N‐cadherin, Vimentin and matrix metalloproteinase‐9 in Calu‐3 cells, whereas overexpression of STRIP2 had the opposite effect in SPC‐A1 cells. Our data indicate that STRIP2 promotes the proliferation and motility of LUAD cells, and this may be mediated through the regulation of the Akt/mTOR pathway and epithelial–mesenchymal transition. These results may facilitate the development of therapeutic strategies to treat LUAD.

## Graphical abstract

Striatin‐interacting protein 2 has been reported to regulate tumor cell growth and migration. Our data indicate that Striatin‐interacting protein 2 promotes the proliferation, invasion and migration of lung adenocarcinoma cells, and this may be mediated through regulation of the Akt/mTOR pathway and epithelial–mesenchymal transition.

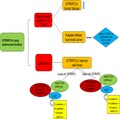

**Abbreviations**
CCK‐8: Cell Counting Kit‐8
EMT: epithelial–mesenchymal transition
LUAD: lung adenocarcinoma
MMP‐9: matrix metalloproteinase‐9
p‐mTOR: phosphorylated-mammalian target of rapamycin
qRT‐PCR: quantitative RT‐PCR
STRIP2: striatin‐interacting protein 2
STRIPAK: striatin‐interacting phosphatase and kinase
TCGA: The Cancer Genome Atlas

Lung cancer, with 2.1 million new cases and 1.8 million new deaths forecasted in 2018, is the prime cause of tumor occurrence and fatalities around the world 1, 2. Lung adenocarcinoma (LUAD), accounting for ~40% of lung cancer cases, is the most widespread form of lung cancer 3. Although bronchoscopy and computed tomography are often used to diagnose lung cancer and LUAD, these methods have certain limitations 4. The low detection rate of LUAD in early stage is the main barrier to the recovery of patients 5. Although cancer treatment has made some progress, especially in immunotherapy and molecular‐targeted therapy, the prognosis of patients with LUAD is still poor, and its 5‐year relative survival rate is no more than 1% 6. Therefore, to perform latent diagnosis in the early stage of LUAD, further studies are warranted to develop the deep mechanism of LUAD, including identifying molecular characteristics associated with patient survival, which may contribute to gene‐targeted therapy.

Striatin‐interacting phosphatase and kinase (STRIPAK) complexes show many physiological functions as a whole or as an individual component, which are highly conserved both in fungal and in mammalian eukaryotes 7, 8. Increasingly, dysregulation of STRIPAK components is involved in various diseases including cancer 9. Striatin‐interacting protein 2 (STRIP2), as one member of the STRIPAK complex and also called Fam40b, took part in regulating tumor cell growth and migration 10, 11. According to the research by Madsen *et al*. 11, STRIP2 ablation lessened cell migration of breast cancer cells. Moreover, evidence of one study indicated that PC3 prostate cancer cell migration was also decreased by silencing STRIP2 12. Up to now, there has been no report on the role of STRIP2 in LUAD growth, migration and the underlying mechanisms.

Herein, we discovered that STRIP2 was highly expressed in patients with LUAD, and high expression of STRIP2 was involved in worse overall survival in LUAD. Intriguingly, we disclosed that ablation of STRIP2 restrained the proliferation, invasion and migration of Calu‐3 cells, and overexpression of STRIP2 accelerated the proliferation, invasion and migration of SPC‐A1 cells, which all might be modulated by the Akt/mTOR pathway and epithelial–mesenchymal transition (EMT). These findings will provide a probable blueprint for the detection and treatment of LUAD.

## Materials and methods

### LUAD patient samples

In this study, the lung cancer RNA sequencing expression data were acquired from The Cancer Genome Atlas (TCGA) database (https://cancergenome.nih.gov/; TCGA‐LUAD Project). There were 535 LUAD patient samples and 59 normal samples, a total of 594 samples for analysis, which are listed in Table S1. The chi‐square test statistical analysis method was used to evaluate the correlation between gene expression and clinical features. The clinical features were grouped to analyze the relationship between the expression of STRIP2 gene and clinical features. The survival analysis was analyzed by the Kaplan–Meier method, and the difference between the two groups was analyzed by log rank test. To investigate whether STRIP2 can be used as an independent predictor of LUAD prognosis, we performed Cox regression analysis on the samples. The data source for clinical correlation, survival and Cox regression analysis is presented in Table S2. 

### Cell preparation

Human LUAD cell lines (NCI‐H1395, SPC‐A1, NCI‐H2009, A549 and Calu‐3) and normal lung cell line (BEAS2B) were acquired from American Type Culture Collection (ATCC, Rockefeller, MD, USA). The cells were maintained in RPMI 1640 medium with 10% FBS, 0.1 mg·mL^−1^ streptomycin and 100 U·mL^−1^ penicillin at 37 °C and 5% CO_2_ incubator.

### Cell transfection

When the cell confluence in six‐well plates reached 80%, the antibiotic‐free medium was replaced 2 h before transfection, then transfection sequences—silencing (si)‐con, 5′‐AATTCTCCGAACGTGTCACGT‐3′; si‐STRIP2#1, 5′‐GTGTACAGCCTTCCGC‐3′; and si‐STRIP2#2: 5′‐CCCCTAAAGCAGCACA‐3′—were transfected into Calu‐3 cells using the Lipofectamine 2000 transfection kit, respectively. The empty vector pcDNA3.1 and overexpression vector pcDNA3.1‐STRIP2 were obtained from Shanghai GenePharma Co., Ltd. (Shanghai, China); then they were transfected into SPC‐A1 cells according to the Lipofectamine 2000 transfection kit. After the cells were incubated in the incubator for 6 h, the complete medium was replaced. The expression of the transferred gene can be observed after 48 h.

### qRT‐PCR assay

The TRIzol reagent (Invitrogen, Carlsbad, CA, USA) was used to extract total RNA of SPC‐A1 and Calu‐3 cells. A total of 1 µg extracted RNA was then reverse transcribed into cDNA using a reverse transcriptase kit (Takara, Dalian, China). Using Power SYBR Green PCR Kit (Applied Biosystems, Foster City, CA, USA), we executed qRT‐PCR to quantify the expressions of STRIP2, along with an internal control of GAPDH. Finally, melting curves were analyzed. Primers used were as follows: STRIP2, F 5′‐AAAGAAGGTCCTGCTCCTGC‐3′, R 5′‐GGCCTGGGTAATCCAACCAA‐3′; GAPDH, F 5′‐ACACCCACTCCTCCACCTTT‐3′, R 5′‐TTACTCCTTGGAGGCCATGT‐3′.

### Western blot

Cells were directly lysed in radioimmunoprecipitation assay lysis buffer. A total of 20 µg protein was separated using 10% SDS/PAGE. Then the proteins were transferred onto a poly(vinylidene difluoride) membrane. After being enclosed with 5% defatted milk at room temperature for 1 h, the membrane was then hatched with the primary antibodies at 4 °C overnight and the secondary antibodies at 37 °C for 2 h. They were visualized by using enhanced chemiluminescence reagents. Western blot data were carried out in three independent experiments.

### Cell Counting Kit‐8

Cell proliferation was assessed by Cell Counting Kit‐8 (CCK‐8; CWBio, Bejing, China) according to the manufacturer's guidelines. Survival curves were generated, and OD_450_ values were derived using graphpad prism v7 (GraphPad Software, San Diego, CA, USA).

### Transwell assays

To explore the influence of STRIP2 on SPC‐A1 and Calu‐3 cell migration and invasion, we conducted Transwell assays. Cell suspension was prepared after 24‐h transfection, and 1 × 10^5^ cells were added to the upper chamber covered with (Transwell invasion assay) or without (Transwell migration assay) Matrigel. Six hundred microliters of complete medium was filled into the lower chamber. After being cultivated at 37 °C overnight, the cells were stained by 0.1% crystal violet dye. Finally, the invaded or migrated cells were photographed using a microscope. The cell numbers were counted in five random fields.

### Colony‐forming assay

The log‐phase cells were trypsinized and pipetted into individual cells to make cell suspensions and then counted. The 60‐mm dish containing 5 mL of 37 °C prewarmed medium was inoculated at a gradient density of 4000 cells per dish and gently rotated to evenly disperse the cells. After that, the cells were incubated in a culture incubator at 37 °C with 5% CO_2_ and saturated humidity for 1–2 weeks. When the macroscopic clones appeared in the culture dish, the culture was terminated. Discarding the supernatant, the precipitate was washed twice with PBS. The cells were fixed by 4% paraformaldehyde for 30 min. Then removing the fixing solution, the cells were stained by 0.1% crystal violet for half an hour. Finally, the clones were counted.

### Statistical analysis

The experimental data were analyzed by spss 22.0 statistical (IBM Corporation, Chicago, IL, USA) and graphpad prism 7.0 analysis software. The comparison of the two groups was performed by Student's *t*‐test. The mean comparison among multiple samples was analyzed by one‐way ANOVA and *post hoc* Bonferroni test. A *P*‐value < 0.05 was considered statistically significant.

## Results

### STRIP2 is highly expressed in patients with LUAD, and high expression of STRIP2 involves worse overall survival in LUAD

**Figure 1:**
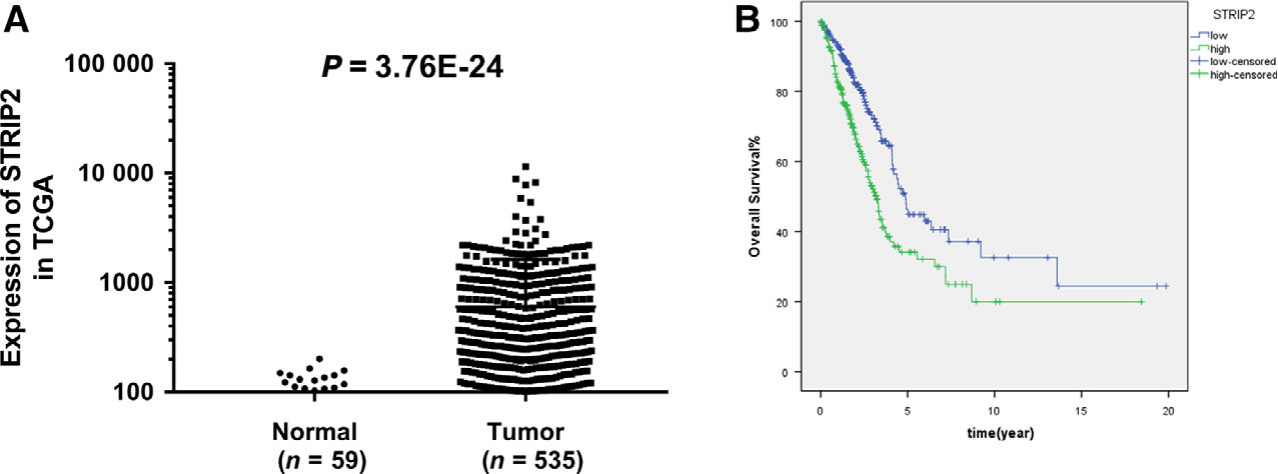
High expression of STRIP2 predicts poor prognosis in LUAD. (A) The mRNA expression level of STRIP2 in LUAD tissue and normal samples. (B) The Kaplan–Meier test was used to analyze the overall survival of STRIP2 in patients with LUAD. The lung cancer RNA sequencing expression data were acquired from TCGA database (TCGA‐LUAD Project).

In this paper, the differential expression of STRIP2 between patients with LUAD and normal samples was investigated by using data acquired from TCGA database. It was indicated that STRIP2 was significantly up‐regulated in patients with LUAD relative to that in normal samples (Fig. 1A, *P* = 3.76E−24). According to the median value of STRIP2 expression, patients were distributed into high‐ and low‐expression groups. The curves revealed that patients with high expression of STRIP2 had a poor overall survival compared with those with low expression of STRIP2 (Fig. 1B, *P* = 0.000). We also found that STRIP2 expression was associated with gender, pathological stage and lymph node metastasis in patients with lung cancer, which was statistically significant (Table 1). More interestingly, in patients with higher‐grade tumors, the STRIP2 expression level was higher. Univariate analysis demonstrated that STRIP2 expression, clinical stage, Pathological‐T, Pathological‐M and Pathological‐N were obviously related to overall survival of patients with LUAD. Multivariate analysis hinted that STRIP2 can be used as an independent predictor of LUAD prognosis (*P < 0.05; Table 2).

**Table 1:** The correlation between STRIP2 expression and the clinicopathological factors of patients with LUAD. The lung cancer RNA sequencing expression data were acquired from TCGA database (TCGA‐LUAD Project).

Characteristics	Expression of STRIP2 (*n*)		*P*‐value
	Low	High	
Age (years)			
< 60	57	79	0.026*
≥ 60	188	166	
Gender			
Female	153	116	0.001*
Male	97	133	
Pathological stage			
I + II	202	183	0.046*
III + IV	44	62	
Pathological‐T			
T1 + T2	220	213	0.345
T3 + T4	28	35	
Pathological‐N			
N0	169	154	0.091
N1	73	92	
Pathological‐M			
M0	164	167	0.456
M1	10	14	

* *P* < 0.05.

**Table 2:** Cox proportional hazards model analysis of clinicopathological features for overall survival in patients with LUAD. The lung cancer RNA sequencing expression data were acquired from TCGA database (TCGA‐LUAD Project). CI, confidence interval; HR, hazard ratio.

Variables	Univariate analysis			Multivariate analysis		
	*P*‐value	HR	95% CI	*P*‐value	HR	95% CI
STRIP2 expression (low/high)	0.000**	1.811	1.345–2.437	0.001*	1.835	1.286–2.619
Clinical stage (I + II/III + IV)	0.000**	2.554	1.874–3.480	0.218	1.367	0.832–2.247
Pathological‐T (T1 + T2/T3 + T4)	0.000**	2.293	1.565–3.359	0.028*	1.703	1.060–2.735
Pathological‐M (M0/M1)	0.006**	2.119	1.237–3.630	0.407	1.314	0.689–2.505
Pathological‐N (N0/N1 + N2 + N3)	0.000**	2.559	1.903–3.442	0.001*	2.004	1.343–2.990
Age (< 60/≥ 60 years)	0.746	1.056	0.760–1.468			
Gender (female/male)	0.667	1.066	0.796–1.427			

* *P* < 0.05

** *P* < 0.01.

### Overexpression and knockdown of STRIP2 in LUAD cell lines

**Figure 2:**
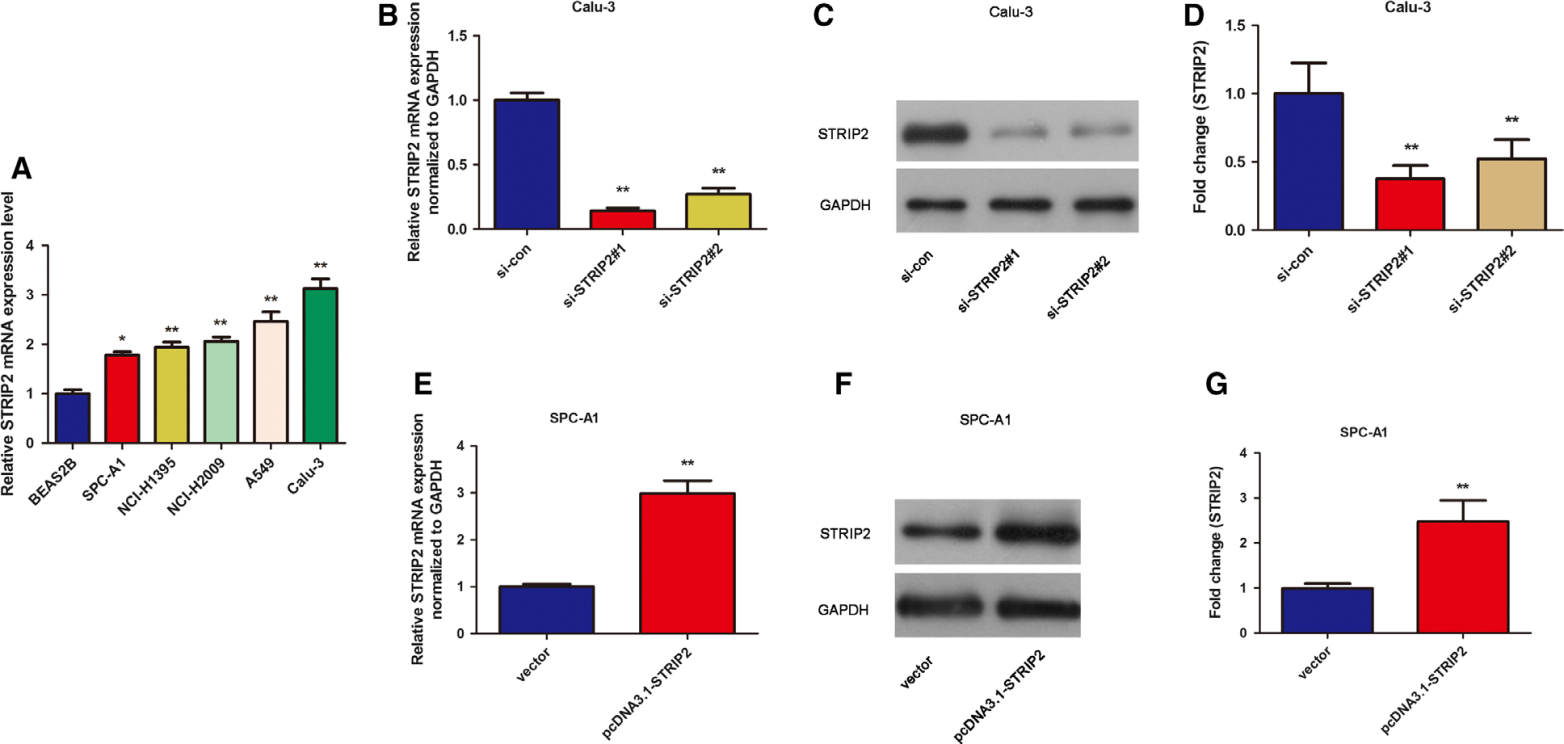
The expression of STRIP2 in LUAD cell lines. (A) The expression of STRIP2 in BEAS2B, NCI‐H1395, SPC‐A1, NCI‐H2009, A549 and Calu‐3 cell lines. *P < 0.05 versus BEAS2B group; **P < 0.01 versus BEAS2B group, one‐way ANOVA. (B) The mRNA expression of STRIP2 in Calu‐3 cells. **P < 0.01 versus si‐control group, one‐way ANOVA. (C, D) The protein levels of STRIP2 in Calu‐3 cells. **P < 0.01 versus si‐control group, one‐way ANOVA. (E) The mRNA expression of STRIP2 in Calu‐3 cells. **P < 0.01 versus vector group, Student's *t*‐test. (F, G) The protein levels of STRIP2 in Calu‐3 cells. **P < 0.01 versus vector group, Student's *t*‐test. Error bars represent ± standard deviation of three independent experiments.

We further studied the expression of STRIP2 in LUAD cell lines. BEAS2B is a normal lung cell line that we used to compare with five different LUAD cell lines: NCI‐H1395, SPC‐A1, NCI‐H2009, A549 and Calu‐3. A significant overexpression of STRIP2 was found in these five LUAD cell lines compared with the BEAS2B cell line. Furthermore, STRIP2 mRNA expression was relatively higher expressed in Calu‐3 cells and lower expressed in SPC‐A1 cells compared with other LUAD cells tested (Fig. 2A). Therefore, in the following assays, detection of STRIP2 knockout effect was performed in the Calu‐3 cell line. Conversely, detection of the influences of STRIP2 overexpression was performed in the SPC‐A1 cell line. The si‐STRIP2 was transfected into the Calu‐3 cell line, and the nonspecific sequence si‐control was transfected as a control. After 48‐h transfection, the total RNA and protein were extracted and detected by qRT‐PCR and western blot. As illustrated in Fig. 2B–D, si‐STRIP2#1 and si‐STRIP2#2 could significantly lessen the expression of STRIP2 both in mRNA and in protein levels in Calu‐3 cells. Also, compared with si‐STRIP2#2, si‐STRIP2#1 is beneficial to the follow‐up experiment. Furthermore, as shown in Fig. 2E–G, pcDNA3.1‐STRIP2 could significantly elevate the expression of STRIP2 both in mRNA and in protein levels in SPC‐A1 cells.

### Down‐regulation of STRIP2 restrains the proliferation of Calu‐3 cells, whereas overexpression of STRIP2 accelerates the proliferation of SPC‐A1 cells

**Figure 3:**
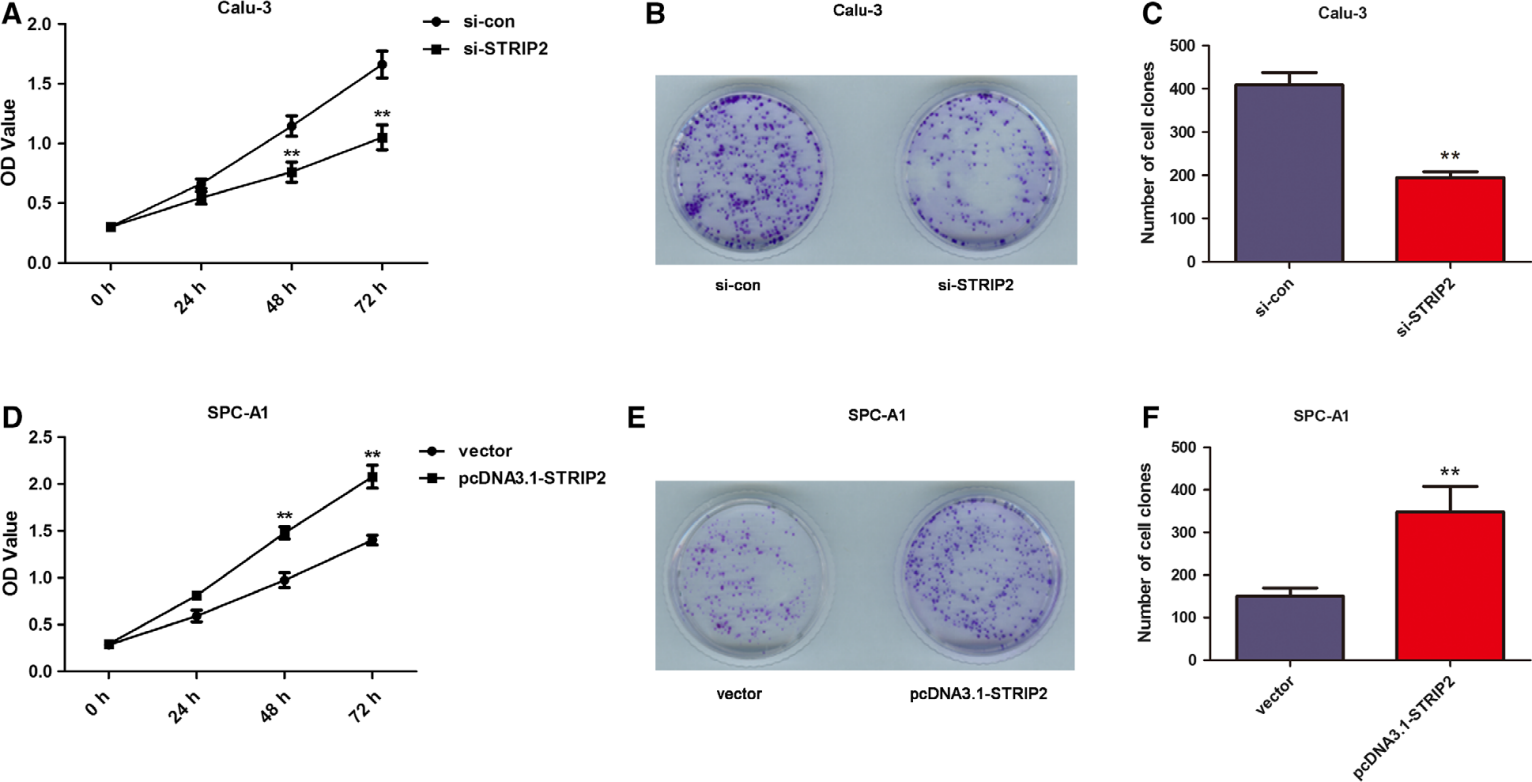
STRIP2 depletion restrained the proliferation of Calu‐3 cells, and overexpression of STRIP2 accelerates the proliferation of SPC‐A1 cells. (A) CCK‐8 analysis insinuated that silencing of STRIP2 restrained cell proliferation in Calu‐3 cells. **P < 0.01 compared with si‐con group, Student's *t*‐test. (B, C) The analysis of colony formation rates in si‐con and si‐STRIP2 groups. **P < 0.01 compared with si‐con group, Student's *t*‐test. (D) CCK‐8 analysis indicated that the overexpression of STRIP2 facilitated cell proliferation in SPC‐A1 cells. **P < 0.01 compared with vector group, Student's *t*‐test. (E, F) Histological analysis of colony formation rates in vector and pcDNA3.1‐STRIP2 groups. **P < 0.01 compared with vector group, Student's *t*‐test. Error bars represent ± standard deviation of three independent experiments.

To investigate the effect of STRIP2 on the proliferation of LUAD cells, we knocked down the expression of STRIP2 in Calu‐3 cells by transfection with si‐STRIP2, and the expression of STRIP2 was overexpressed in SPC‐A1 cells by transfection with pcDNA3.1‐STRIP2. STRIP2 knockdown could sharply reduce the proliferation in Calu‐3 cells after being cultured for 48 and 72 h, but there was no significant effect at 24 h, as revealed by CCK‐8 assay (*P* < 0.01; Fig. 3A). The clone formation abilities of Calu‐3 cells were obviously restrained by silencing of STRIP2 when compared with si‐con (*P* < 0.01; Fig. 3B,C). As displayed in Fig. 3D, the results indicated that the OD_450_ value of SPC‐A1 cells increased in the pcDNA3.1‐STRIP2 group compared with the vector group, proving that overexpression of STRIP2 accelerated SPC‐A1 cell proliferation. Moreover, the clone formation abilities of SPC‐A1 cells were boosted after overexpression of STRIP2 when compared with the vector group (*P* < 0.01; Fig. 3E,F). All of these earlier findings insinuated that STRIP2 depletion had a repressive effect and STRIP2 overexpression had a stimulative effect on the growth of LUAD cells.

### Knockdown of STRIP2 suppresses cell invasion and migration in Calu‐3 cells, and overexpression of STRIP2 augments cell invasion and migration in SPC‐A1 cells

**Figure 4:**
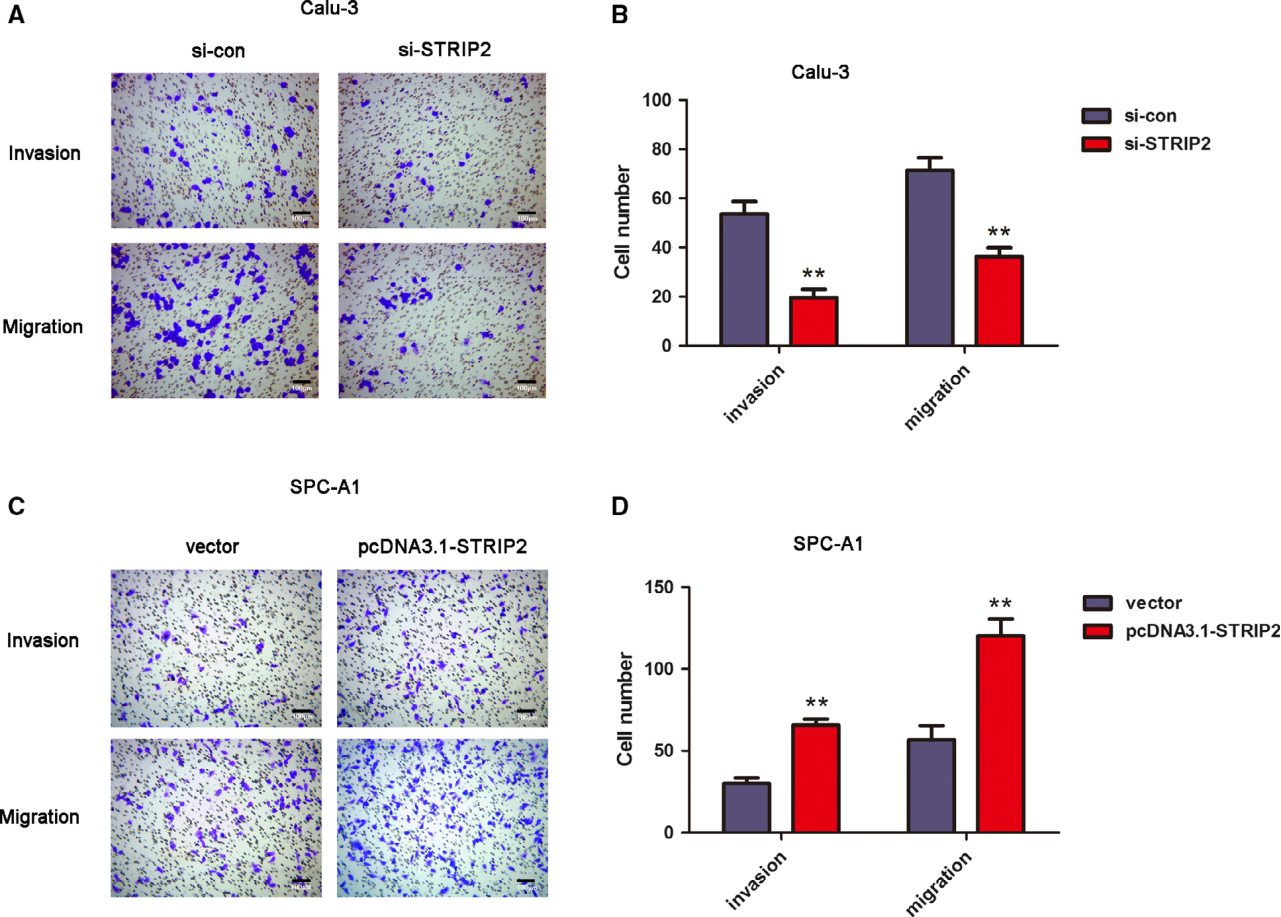
Depletion of STRIP2 suppressed the migration and invasion of Calu‐3 and SPC‐A1 cells. (A) Bright‐field images of Transwell assay in Calu‐3 cells were acquired by an inverted microscope (scale bars, 100 µm). (B) Quantification of (A). **P < 0.01 versus si‐control group, Student's *t*‐test. (C) Bright‐field images of Transwell assay in SPC‐A1 cells were acquired by an inverted microscope (scale bars, 100 µm). (D) Quantification of (C). **P < 0.01 versus vector group, Student's *t*‐test. Error bars represent ± standard deviation of three independent experiments.

The influence of STRIP2 on the invasion and migration of the LUAD cell lines Calu‐3 and SPC‐A1 was investigated using Transwell assays. Silencing of STRIP2 significantly reduced the number of crystal violet‐stained Calu‐3 cells in the invasion and migration assays (Fig. 4A,B). On the contrary, overexpression of STRIP2 significantly fortified the number of crystal violet‐stained SPC‐A1 cells in Transwell assay (Fig. 4C,D). These consequences suggested that silencing of STRIP2 inhibited cell invasion and migration in Calu‐3 cells, and overexpression of STRIP2 augmented cell invasion and migration in SPC‐A1 cells.

### STRIP2 can modulate the activation of the Akt/mTOR pathway in Calu‐3 and SPC‐A1 cells

**Figure 5:**
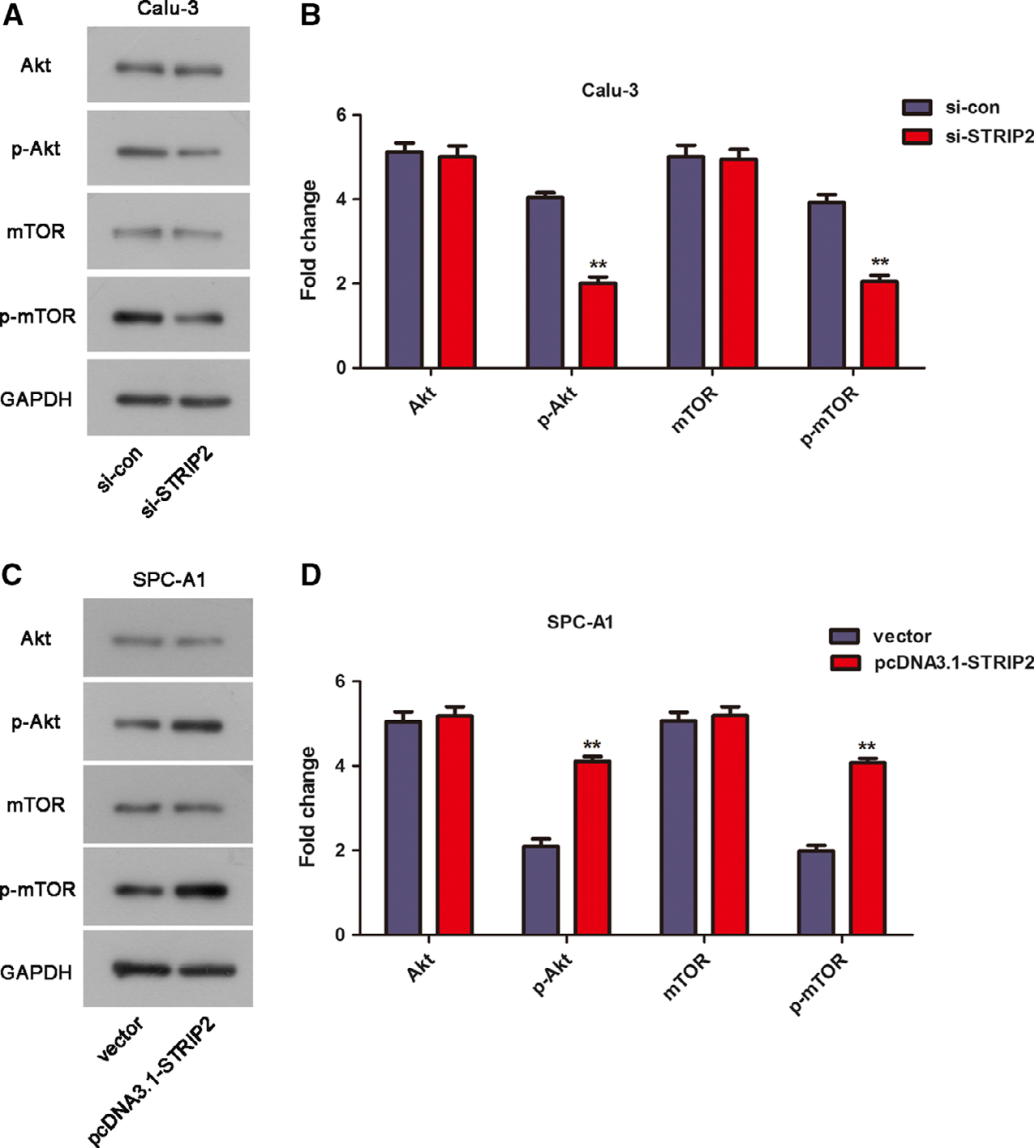
STRIP2 modulated the activation of the Akt/mTOR pathway in Calu‐3 and SPC‐A1 cells. (A, B) The expression of Akt, p‐Akt, mTOR and p‐mTOR in Calu‐3 cells. **P < 0.01 versus si‐control group, Student's *t*‐test. (C, D) The expression of Akt, p‐Akt, mTOR and p‐mTOR in SPC‐A1 cells. **P < 0.01 versus vector group, Student's *t*‐test. Error bars represent ± standard deviation of three independent experiments.

To gain a deeper understanding of the mechanism by which STRIP2 affects cell proliferation of Calu‐3 and SPC‐A1 cells, we tested the protein expression levels of Akt, p‐Akt, mTOR and phosphorylated‐mammalian target of rapamycin (p‐mTOR) by western blot. Figure 5A,B illustrated that STRIP2 ablation distinctly decreased the protein levels of p‐Akt and p‐mTOR in Calu‐3 cells. Inversely, overexpression of STRIP2 obviously enhanced the protein levels of p‐Akt and p‐mTOR in SPC‐A1 cells (Fig. 5C,D). Above all, these outcomes demonstrated that STRIP2 exerted its function on LUAD cell proliferation partially through the Akt/mTOR pathway.

### STRIP2 can regulate EMT in Calu‐3 and SPC‐A1 cells

**Figure 6:**
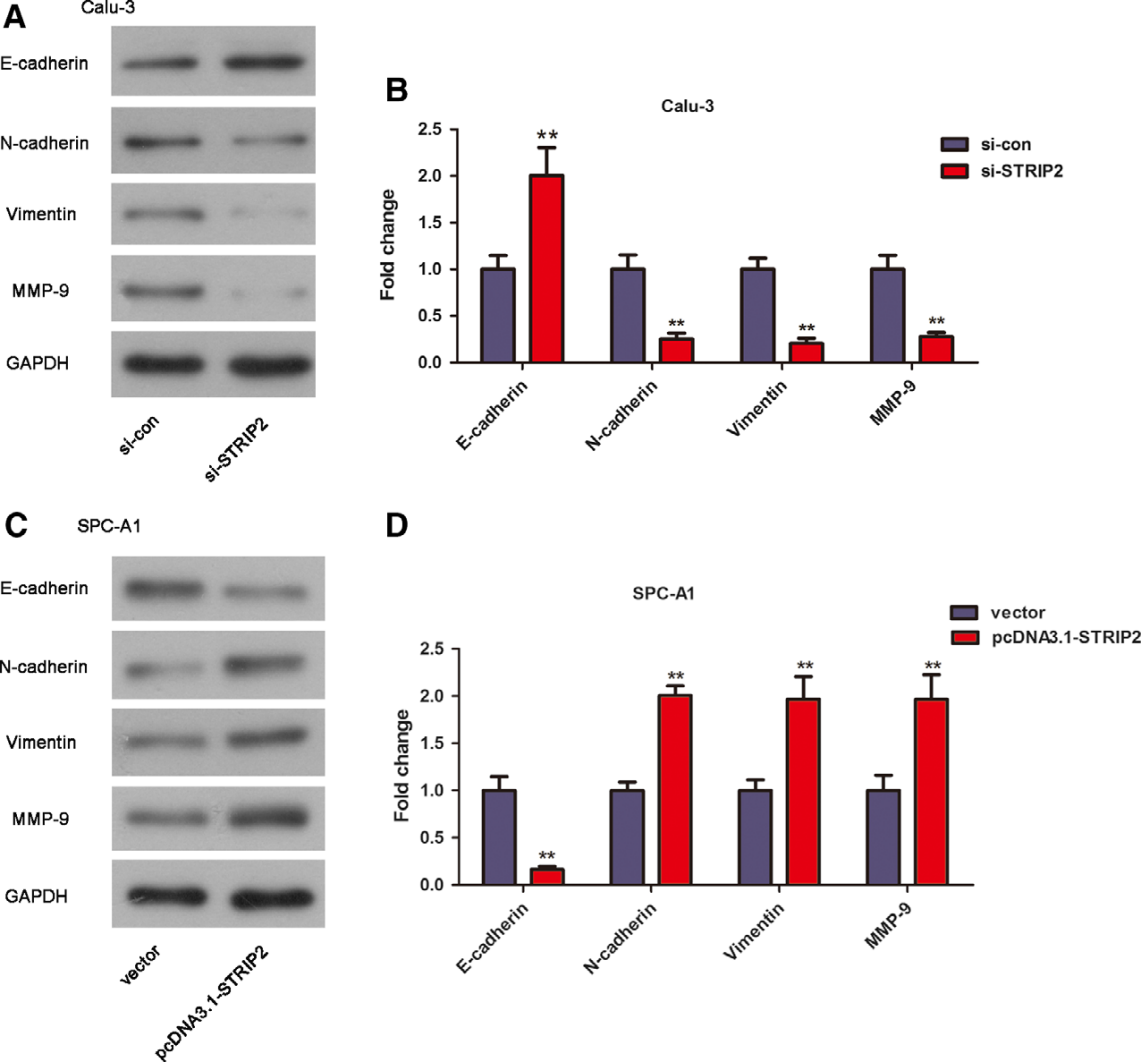
Silencing of STRIP2 regulated EMT in Calu‐3 cells, and overexpression of STRIP2 regulated EMT in SPC‐A1 cells. (A, B) The expression of E‐cadherin, N‐cadherin, Vimentin and MMP‐9 in Calu‐3 cells. **P < 0.01 versus si‐control group, Student's *t*‐test. (C, D) The expression of E‐cadherin, N‐cadherin, Vimentin and MMP‐9 in SPC‐A1 cells. **P < 0.01 versus vector group, Student's *t*‐test. Error bars represent ± standard deviation of three independent experiments.

EMT is critical for cell motility of LUAD, and E‐cadherin, N‐cadherin, Vimentin and matrix metalloproteinase‐9 (MMP‐9) are the markers of EMT. To gain a deeper understanding of the mechanism by which STRIP2 affects cell motility of Calu‐3 and SPC‐A1 cells, we tested the protein expression levels of E‐cadherin, N‐cadherin, Vimentin and MMP‐9 by western blot. As shown in Fig. 6A,B, the protein level of E‐cadherin was significantly heightened to 2.01‐fold of the si‐con group after silencing of STRIP2. Knockdown of STRIP2 significantly lessened the protein expression levels of N‐cadherin, Vimentin and MMP‐9 to 0.25‐, 0.20‐ and 0.28‐fold of the si‐con group. Moreover, overexpression of STRIP2 obviously receded the protein expression levels of E‐cadherin to 0.16‐fold of the si‐con group. Nevertheless, the protein levels of N‐cadherin, Vimentin and MMP‐9 were significantly enhanced to 2.01‐, 1.97‐ and 1.96‐fold of the vector group after overexpression of STRIP2 (Fig. 6C,D).

## Discussion

Based on reported studies, we are the first to report the role of STRIP2 on LUAD. To be specific, we disclosed that high expression of STRIP2 was related to poor prognosis of patients with LUAD. Furthermore, knockdown of STRIP2 significantly restrained cell proliferation, invasion and migration possibly by inactivating the Akt/mTOR pathway and EMT in Calu‐3 cells, whereas STRIP2 overexpression significantly promoted cell proliferation, invasion and migration, which might activate the Akt/mTOR pathway and EMT in SPC‐A1 cells. Therefore, STRIP2 may be a novel promising target for LUAD treatment.

Our outcomes manifested that high expression of STRIP2 was related to poor prognosis of patients with LUAD. Growing evidence demonstrated that STRIP2 regulated STRIPAK complex activity by interacting with its key core striatin 13. STRIP2 is a member of STRIPAK complex that may be concerned with modulating cell proliferation, migration, adhesion and so on 14. Hence, STRIP2 may have significant effects in several biological courses, including cell growth, differentiation, and development of vascular and neural function of cardiac disease and cancer. Madsen *et al*. 11 hold the view that STRIP2 had a crucial role in tumor development and metastasis. It has been reported that MDA‐MB231 breast cancer cell migration was decreased by STRIP2 deficiency 11. Besides, according to a recent study, silencing of STRIP2 also decreased cell migration in prostate cancer 12. To date, whether STRIP2 has a role in LUAD proliferation, migration and the underlying mechanisms has not been reported. In our paper, we constructed Calu‐3 cells with down‐regulated STRIP2 and SPC‐A1 cells with overexpressed STRIP2. We revealed for the first time that knockdown of STRIP2 significantly repressed cell proliferation, invasion and migration, whereas STRIP2 overexpression significantly facilitated cell proliferation, invasion and migration in LUAD cells.

In our study, we discovered that the influence of STRIP2 on LUAD cell proliferation might be activated by the Akt/mTOR pathway. In controlling cell survival, differentiation and malignant transformation, the AKT/mTOR pathway is one of the main pathways 15. Suppression of this pathway is considered a latent antitumor therapy 16. The Akt/mTOR pathway is a critical pathway for LUAD cell proliferation 15, 17. Through the Akt/mTOR pathway, xeroderma pigmentosum complementation group C repression rescued cisplatin resistance in LUAD cells 17. However, a few articles reported that STRIP2 was involved in the modulation of the Akt/mTOR pathway. The deficiency of STRIP2 has been illustrated to restrain the proliferation and migration of vascular smooth muscle cells through affecting the Akt pathway 10. In line with the earlier reports, our article manifested that overexpression of STRIP2 promoted cell proliferation, which might be achieved by the activation of the Akt/mTOR pathway in LUAD cells.

In our article, we also found that the impact of STRIP2 on LUAD cell invasion and migration was possibly regulated by EMT. EMT is a vital process in embryonic upgrowth and tumor migration 18, 19. The invasion and migration ability of tumor cells are obviously heightened after the incidence of EMT 20. Through targeting the PI3K/Akt pathway, CNTN‐1 boosted chemoresistance in LUAD via abduction of EMT 21. Studies have indicated that the expression levels of some vital factors (such as E‐cadherin, N‐cadherin, Vimentin and MMP‐9) have been detected during the process of EMT 22, 23. As reported, E‐cadherin is a suppressor, whereas N‐cadherin, Vimentin and MMP‐9 are promoters 24, 25. E‐cadherin can serve as a major conditioning agent in epithelial cell adhesion 26. Using a mouse model, ablation of E‐cadherin facilitated cancer progression and metastasis in LUAD 27. N‐cadherin, which is an adhesion molecule of EMT, was first identified in the nervous system 28. As reported, depletion of N‐cadherin suppressed cell invasion and migration in LUAD 29. As a biomarker of EMT, Vimentin is an intermediate filament protein that plays a role in cell migration to keep structure and motility 30. Through maintaining heterotypic cancer cell–cancer‐associated fibroblast interactions during collective invasion, Vimentin was required for LUAD metastasis 30. MMPs, which can proteolyze all elements of matrix in extracellular, are a big class of endopeptidases 31. MMP‐9, as one member of the MMP family, can be used as a controller for tumor neovascularization 32. The effect of MMP‐9 on cancer growth is achieved by type IV collagen degradation (the main constituent part of the basement membrane) 33. Wen and Li 34 indicated that MMP‐9 was highly expressed in LUAD. In our findings, we identified that overexpression of STRIP2 obviously receded the protein expression levels of E‐cadherin and enhanced the protein levels of N‐cadherin, Vimentin and MMP‐9 in LUAD, suggesting that STRIP2 exhibited a promoting effect on the EMT of LUAD cells.

## Conclusions

Taken together, we argue for the first time that STRIP2 was highly expressed in patients with LUAD, and high expression of STRIP2 was involved in worse overall survival in LUAD. In addition, knockdown of STRIP2 significantly suppressed cell proliferation, invasion and migration in Calu‐3 cells, whereas STRIP2 overexpression significantly promoted cell proliferation, invasion and migration in SPC‐A1 cells, which might be modulated by the Akt/mTOR pathway and EMT. Our observations will provide novel insight into the regulation of STRIP2 during the pathogenesis of LUAD and have important clinical implications for patients with LUAD. However, more *in vivo* studies are needed to further verify our results.

## Conflict of interest

The authors declare no conflict of interest.

## Author contributions

L‐MQ and H‐TM designed this work. L‐MQ, Y‐HS, T‐TC and J‐JC conducted the experiments. L‐MQ wrote the paper. H‐TM revised the paper. All the authors approved the final version of the paper.

## Supporting information


**Table S1.** Analyzed TCGA samples.

**Table S2.** Data source for clinical correlation, survival and Cox regression analysis.
